# Review of autism spectrum disorder databases for the identification of candidate genes

**DOI:** 10.1093/database/baaf067

**Published:** 2025-10-15

**Authors:** Diana Martínez-Minguet, René Noel, Alberto García S., Mireia Costa, Oscar Pastor

**Affiliations:** PROS Group, Valencian Research Institute for Artificial Intelligence (VRAIN), Universitat Politècnica de València, Camí de Vera s/n, Valencia 46022, Spain; PROS Group, Valencian Research Institute for Artificial Intelligence (VRAIN), Universitat Politècnica de València, Camí de Vera s/n, Valencia 46022, Spain; Escuela de Ingeniería Informática, Facultad de Ingeniería, Universidad de Valparaíso, General Cruz 222, Valparaíso 2362905, Chile; PROS Group, Valencian Research Institute for Artificial Intelligence (VRAIN), Universitat Politècnica de València, Camí de Vera s/n, Valencia 46022, Spain; PROS Group, Valencian Research Institute for Artificial Intelligence (VRAIN), Universitat Politècnica de València, Camí de Vera s/n, Valencia 46022, Spain; PROS Group, Valencian Research Institute for Artificial Intelligence (VRAIN), Universitat Politècnica de València, Camí de Vera s/n, Valencia 46022, Spain

## Abstract

Research into the genetics of autism spectrum disorder (ASD) seeks to unravel its complex genetic background by identifying genes associated with the condition at varying levels of confidence. While these findings hold significant potential for clinical applications, the dispersed nature of scientific evidence presents a challenge for the reliable identification of ASD candidate genes. Although ASD candidate genes are gathered in genetic databases, these vary widely in the gene sets, biological information, and confidence level classification methods, leading to inconsistencies and complicating research efforts. This study aims to identify and assess the quality and reliability of ASD genetic databases to support more robust identification of ASD candidate genes. Using a Systematic Mapping Study, we identified 13 specialized databases. We then followed a Data Quality Approach in two stages, first assessing Accessibility, Currency, and Relevance dimensions to select the potentially relevant databases to be used as ASD candidate gene sources. The selected databases were analysed, assessing Completeness—at schema and data level—, and Consistency between high-confidence ASD genes. The four selected databases are: AutDB, SFARI Gene, GeisingerDBD, and SysNDD. SFARI Gene demonstrated the highest completeness at schema level (89%), while AutDB showed the highest completeness at data level (90%). However, only 1.5% consistency was observed across the four databases in their classification of high-confidence ASD candidate genes. Our findings highlight the unique contributions of each database and reveal substantial inconsistencies in gene classification, driven by differences in scoring criteria and the scientific evidence considered. These inconsistencies have important implications for both clinical users and researchers, as conclusions may vary depending on the database used. This study supports researchers when using ASD genetic databases, promoting consistent interpretation and improved clinical decisions.

## Introduction

Autism spectrum disorder (ASD) is a neurodevelopmental disorder (NDD) with significant heritability, estimated at up to 80% [1, 2]. Although diagnosed based on behavioural criteria according to the standardized diagnosis manuals, DSM-V [1] and ICD-11 [3], genetic research has highlighted a strong genetic contribution, leading to major advancements in this active field of study [4].

ASD genetics is highly heterogeneous, with both common and rare variants contributing to the disorder [5]. The aggregation of the scientific evidence on variants conferring risk of ASD has allowed the identification of candidate genes that are potentially associated to ASD [6], which are particularly relevant to be used for ASD genetic testing and counselling [7]. Genetic testing for ASD patients is highly recommended due to its significant benefits, including enhancing precision medicine by improving the accuracy of counselling on prognosis and recurrence risk, as well as offering personalized support based on the patient’s genetic information [5, 8].

Given the complexity of ASD genetics and the vast amount of scientific evidence dispersed in the literature, identifying ASD candidate genes is an arduous work. The strength of the association of a gene with ASD can vary depending on the supporting scientific evidence. The strength, i.e. the confidence level, of this association is established according to a scoring method, which considers the scientific evidence from the literature and classifies genes according to a given criteria, in order to represent how strong the association with ASD is. This stratification allows clinicians to easily differentiate between genes with a clear association to ASD (high-confidence genes) and those with less supporting evidence, promoting more accurate ASD genetic testing and counselling for patients and their families.

In this context, specialized databases providing information on association between genes and ASD, such as SFARI Gene and GeisingerDBD, are very useful since they integrate scientific evidence from the literature and provide ASD candidate genes, with the supporting variants or information on biological functions [9–11]. However, as previously noted, scientific evidence in the genomic field is often scattered across the literature, a situation commonly referred to as the genomic data chaos [12]. As a result, databases vary widely in the gene sets, biological information, and scoring methods they provide for ASD candidate genes, leading to inconsistencies and complicating research efforts. It is not a feasible task for clinicians or researchers to manually explore all available resources to spot the ones relevant for genetic testing and counselling. Consequently, most professionals rely on a limited number of familiar databases, often selected for convenience or institutional availability, potentially overlooking important gene–disease associations described elsewhere.

This fragmented and inconsistent landscape has direct clinical repercussions: a diagnosis may be missed, delayed, or misinterpreted simply because a specific gene, variant details or associated syndrome is not reported in the consulted database. For instance, consider the case detailed in [13]. A child with high risk for autism undergoes testing for the MTHFR gene, which reveals a risk variant and leads to tailored treatment with a favourable outcome. While the MTHFR gene and variant are listed in the SFARI Gene database, they are missing from GeisingerDBD. Consequently, an interpretation of the test results derived solely from the latter database would overlook this diagnosis, thereby failing to recommend the necessary treatment for the patient.

In this context, assessing both the completeness (what information is present) and consistency (how information aligns across databases) becomes essential to guide informed decisions in ASD clinical genetics. While previous studies have proposed gene lists or individual curation strategies [14, 15], to the best of our knowledge, no prior work has systematically assessed the structural completeness, data-level coverage, and classification consistency across multiple ASD genomic databases. Our study aims to fill this gap by identifying and assessing the quality and reliability of ASD genetic databases providing ASD candidate genes. To achieve this, we pursue two main goals, (i) to identify all existing specialized databases in the scientific literature providing ASD candidate genes by conducting a Systematic Mapping Study and (ii) to select and analyse the potentially relevant databases to be used as ASD candidate gene sources, by performing a comparative analysis of the databases following a data quality approach.

Through this study, we provide a comprehensive overview of the current landscape of ASD candidate gene databases, highlighting key quality indicators and identifying the most reliable and informative resources. Our findings aim to support researchers and clinicians in making evidence-based choices when using genomic databases for ASD-related investigations, ultimately contributing to more consistent genetic interpretations and improved clinical decision-making.

## Materials and methods

**Figure 1.**
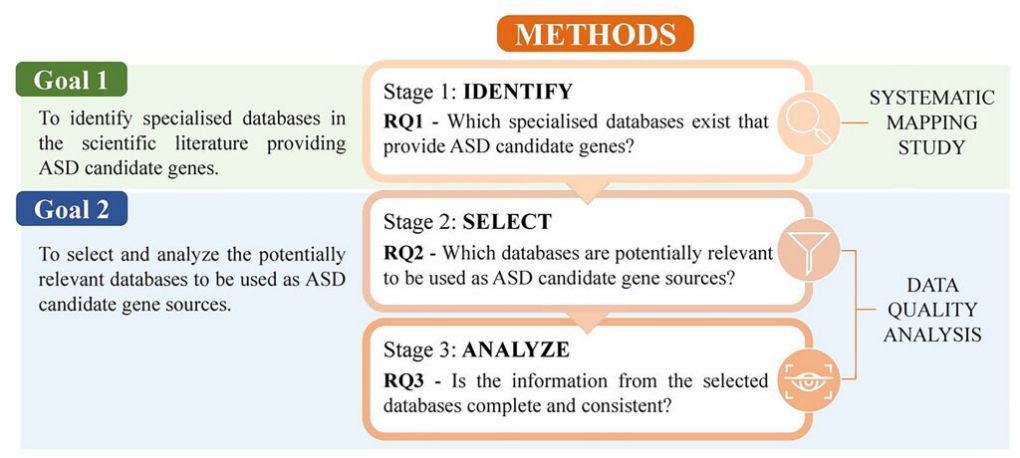
Methods workflow. Methods and research questions addressed in this study.

Our research is structured into three stages, each stage focusing on a different research question (Fig. 1). In the first stage we address RQ1—Which specialized databases exist that provide ASD candidate genes? To answer this RQ, we conduct a Systematic Mapping Study, following the PRISMA-ScR reporting guidelines [16]. This will allow us to achieve the first goal.

To accomplish our second goal we adopt a data quality (DQ) approach. This approach is based on existing procedures of DQ in healthcare settings [17, 18] and will allow us to obtain the quantitative information to achieve the goal proposal. Several DQ dimensions are widely studied in the literature [19]. For this study, we focus on five specific dimensions (Table 1): Accessibility [20, 21], Currency [18, 20], Relevance [18, 21], Completeness [17, 21, 22], and Consistency [17, 23]. The DQ approach is applied in two stages. First, we address RQ2—Which databases are potentially relevant to be used as ASD candidate gene sources?, where we filter the identified databases based on the first three DQ dimensions. Then, we tackle RQ3—Is the information from the selected databases complete and consistent?, where we analyse the data of the selected databases assessing the two remaining DQ dimensions.

**Table 1.** DQ dimensions.

Dimension	General definition
Accessibility	The extent to which the database is available and its data easily and quickly retrievable [21].
Currency	The extent to which the database is up to date [20].
Relevance	The extent to which the database is helpful for the task at hand [21].
Completeness	The extent to which data are of sufficient breadth, depth, and scope for the task at hand [21].
Consistency	The extent to which data elements between different databases agree [23].

The search strategy and the metrics defined to evaluate the DQ dimensions are described in the following sections.

### Stage 1: Identification of databases

Our study focuses on databases specializing in ASD due to their detailed and targeted information on genes and genetic variants associated with autism. Unlike general disease databases that cover various conditions, ASD databases are designed specifically to catalogue ASD-related genetic data. We also include databases specialized in NDDs or brain-related disorders (BRD) to provide a comprehensive search, given the shared genetic backgrounds [24]. This approach will allow us to access a wider range of relevant resources while maintaining a specialized focus. We conduct a Systematic Mapping Study to identify articles that describe specialized databases containing ASD candidate genes based on gathered genetic evidence.

**Figure 2.**
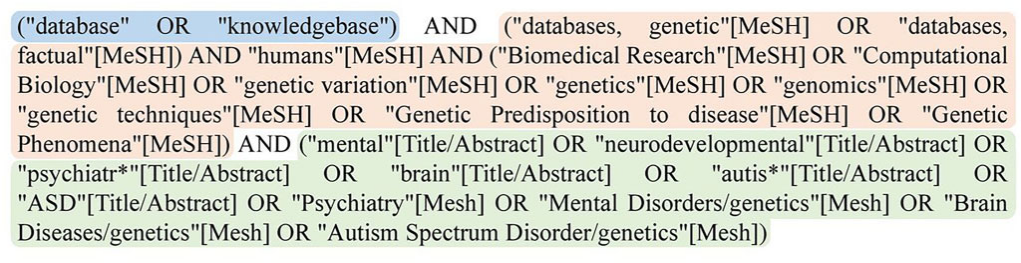
Advanced search filter used to search the PubMed information source.

The information sources consulted include PubMed, ScienceDirect, Scopus, and Web of Science. The same search strategy is applied across all four sources. As PubMed is the most widely recognized repository of biomedical literature, it served as the foundation for constructing the initial search string. We apply an Advanced Search filter using specific keywords and terms from the Medical Subject Headings thesaurus, which is a controlled and hierarchically organized vocabulary produced by the National Library of Medicine used for indexing, cataloguing, and searching of biomedical and health-related information [25]. No restriction is applied as to the year of publication, since a database mentioned in an old publication may still be actively maintained and relevant today. The filter, shown in Fig. 2, consists of three blocks. One block for the explicit mention of ‘database’ or ‘knowledgebase’ terms throughout the article, another block to specify databases related to the genetic domain, and the last block regarding that genetic databases provide information on the disorders of interest. We carried out the broadest search within PubMed and complemented it with searches in Scopus, ScienceDirect, and Web of Science, adapting the string as needed to align with the specific filters of each source (see Supplementary file).

Eligibility criteria are applied to filter the articles retrieved from the search string. Inclusion criteria encompass articles providing a database specialized in ASD, NDD, or BRD, always containing information on ASD genetic association, and articles gathering multiple databases containing at least one database of interest for the present study. Exclusion criteria encompass articles not containing any database or the databases presented not being of interest for our study. We initiate the selection process by screening titles and abstracts, followed by a thorough review of the full articles. This approach will allow us to compile the initial set of chosen articles. We then conduct forward and backward snowballing as described by Wohlin [26], to identify other articles providing new databases or referring to updated versions of previously identified databases. We iterate until no new relevant studies are discovered. The search was done on 17th February 2025.

The resulting set of articles provide all identified databases from the information sources. The databases’ links are extracted from the articles for their analysis.

### Stage 2: Selection of databases

The first step of the DQ approach consists on filtering the databases identified to select only the pertinent ones for the task at hand. The dimensions analysed are Accessibility, Currency, and Relevance.

Accessibility is evaluated by verifying the operability of the database, which involves two metrics: checking the status of the website link (active link) and ensuring that data can be successfully downloaded (downloadable). This dimension is considered verified for a database only if both metrics yield positive results. For the Currency dimension, we assess whether the database content is updated enough for the task at hand (updated). Given the dynamic nature of genetic research, continuous updates are crucial for ensuring database reliability, particularly when the information is intended for clinical use [27]. We consider a 2-year window for the limit update date.

Finally, the Relevance dimension concerns the utility of data for the specific task at hand. In our study, we target clinicians who need to select ASD candidate genes to be used in genetic testing and counselling. Certain databases incorporate a scoring method that classifies ASD candidate genes according to the confidence level of the association between the gene and ASD based on the supporting evidence [28, 29], facilitating the prioritization of the most relevant genes. Therefore, the metric assessed is whether a database includes a scoring method for ASD candidate genes, as this feature enhances its usefulness for clinicians in making informed decisions [14].

Databases that meet all metrics are considered potentially relevant for use as sources of ASD candidate genes and are thus selected for a deeper data analysis.

### Stage 3: Analysis of databases

Following the second step of the DQ approach, the dimensions assessed in this stage are Completeness and Consistency.

Completeness is evaluated at both the schema and data levels. To assess the completeness at schema level (CSL) we identify the relevant fields to be covered by a database specializing in ASD genetic association. The rationale of the selected fields for the genetic testing and counselling context is provided in Table 2. The metric assessed is defined by computing the ratio between the number of covered fields by the database compared to the total number of identified fields:


(1) \begin{eqnarray*}
CLS = \frac{{Num.\,\mathit{covered}\ \mathit{fields}}}{{Num.\,\mathit{total}\ \mathit{fields}}}.
\end{eqnarray*}

**Table 2.** Fields to be covered by a database specialized in ASD.

Field	Rationale
Variant allele change (V); Variant type (V)	ASD candidate genes are identified based on aggregating variants within specific genes. Detailed information about the supporting variants is crucial for database reliability. The identification of previously reported variants can be highly useful for the interpretation of genetic testing results, since a past clinical correlation increases the likelihood that the variant is disease related in the patient [60].
CNV information (V)	Copy number variants (CNVs) are differences in the number of copies of a specific segment of DNA among different individuals’ genomes, which may affect many genes. Studies have found recurrent CNVs in autistic individuals, strongly implicating these variants in ASD pathology and enabling the identification of ASD candidate genes [61, 62]. The identification of a recurrent CNV in an individual’s genome can provide a possible explanation for the patient’s autism [63].
Family type (V)	ASD genetic studies may annotate the family type associated to the variant identified, indicating whether autism occurs in a single individual (simplex) or in multiple individuals within the same family (multiplex). Research on genetic differences between individuals from simplex and multiplex families is crucial for studying risk factors associated with ASD, specially related to the inherited risk [64, 65].
Inheritance (V); parental transmission (V); inheritance pattern (G)	Inheritance specifies if the variant is de novo or inherited. Parental transmission specifies, for the inherited case, if the transmission is maternal, paternal, or both. The specific inheritance pattern is regarded to the gene. Inheritance details allow genetic counselors to provide accurate information to families about the likelihood of passing on the variant to future generations and helps estimate the recurrence risk of ASD within families [66].
Syndromic classification (G)	ASD is clinically classified into syndromic and nonsyndromic forms based on the presence or absence of additional symptoms alongside autism traits, respectively [67]. Although syndromic autism cases are less common, they have contributed significantly to progress in autism genetics, as most syndromic cases have a known genetic cause [68]. Investigating the genetic basis of syndromic autism can enhance genetic testing strategies by facilitating the development of syndrome-specific tests.
Associated disorders or syndromes (G)	Individuals with ASD are more likely to develop MD conditions than the general population [69]. Keeping track of associated disorders for an ASD candidate gene is relevant since it can allow for the identification and treatment or prevention of medical comorbidities [14].

In brackets, we specify if the field is related to the variant reported information (V), or to the gene (G).

Completeness at data level (CDL) is evaluated by comparing the ASD candidate genes provided by each database. Given that some databases contain information on other disorders besides ASD, data is filtered to obtain only the information related to ASD. Since gene symbols evolve with time we employ the HGNC Multi-symbol checker tool [30] to ensure that all databases use the same symbol to refer to the same gene. The metric is defined as the ratio between the number of ASD candidate genes provided by a database with respect to the total number of unique ASD candidate genes from all databases:


(2) \begin{eqnarray*}
CDL = \frac{{Num.\,\mathit{database}\ ASD\ \mathit{candidate}\ \mathit{genes}}}{{Num.\,\mathit{total}\ \mathit{unique}\ ASD\ \mathit{candidate}\ \mathit{genes}}}.
\end{eqnarray*}

The last dimension assessed is Consistency (CNS), where we evaluate whether the information regarding ASD candidate gene classification is consistent among the databases. To do so we consider the scoring method provided by each database. As mentioned in Stage 2: Selection of databases, the scoring method is the most important characteristic because it allows to prioritize the most relevant genes associated to ASD. Since each scoring method is defined differently by each database—different number of levels and different definitions—, we compare the high-confidence (HC) genes, i.e. the ASD candidate genes with the highest score defined by each database. The metric evaluates the ratio between the overlapping HC genes and the total number of unique HC genes provided by all databases:


(3) \begin{eqnarray*}
CNS = \frac{{Num.\,\mathit{overlapping}\ HC\ ASD\ \mathit{candidate}\ \mathit{genes}}}{{Num.\,\mathit{total}\ \mathit{unique}\ HC\ ASD\ \mathit{candidate}\ \mathit{genes}}}.
\end{eqnarray*}

## Results

This section is organized following the three stages of the methods section. We first provide the databases retrieval as a result of the Systematic Mapping Study. Afterwards, a subset of databases is selected according to the assessment of three dimensions of the DQ approach, namely Accessibility, Currency, and Relevance. Finally, for this filtered set of databases, we assess the Completeness and Consistency dimensions.

### Stage 1: Identification of databases

**Figure 3.**
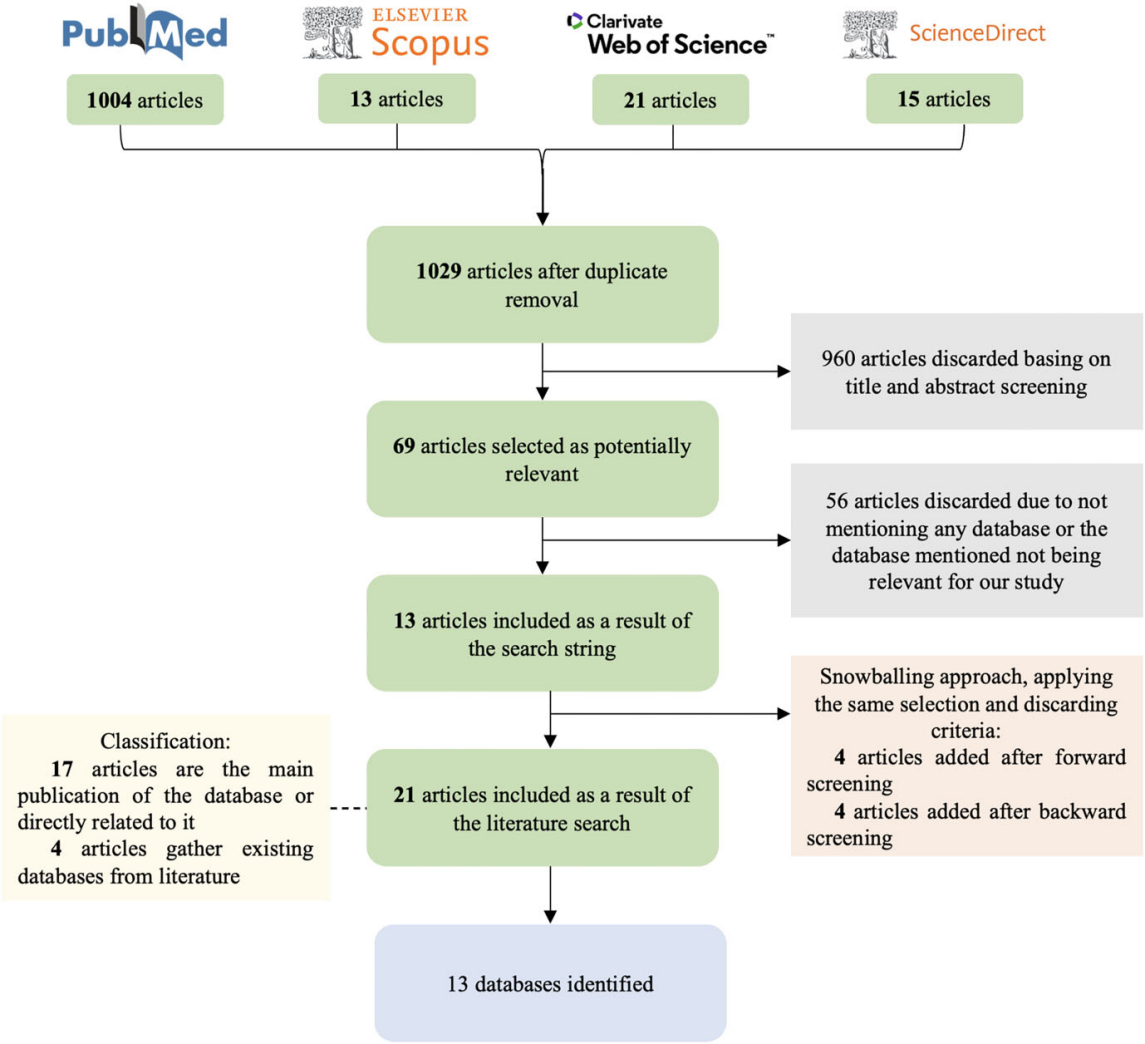
Systematic Mapping Study flow-chart.

To perform the Systematic Mapping Study we followed the flow-chart depicted in Fig. 3. Using advanced search filters, the PubMed query retrieved 1004 records. To ensure comprehensive coverage, this was supplemented with 13 articles from Scopus, 21 from Web of Science, and 15 from ScienceDirect. After removing duplicates, a total of 1029 unique articles were included. Inclusion and exclusion criteria were applied in two steps in order to select the articles providing a database related to ASD, NDD, or BRD genetic information. We first screened all entries filtering by title and abstract, where 960 articles were discarded. The remaining 69 articles were examined in their entirety in order to identify those providing an ASD, NDD, or BRD database containing genetic information. From these 69 studies, 56 were excluded because they either did not provide a database or the database provided did not relate to ASD, NDD, or BRD genetic information. As a result, 13 articles were selected that provided at least one database of interest. After the snowballing procedure, eight additional articles were added, applying the same exclusion and inclusion criteria. We first conducted forward snowballing searching among studies citing our first set of articles and retrieved four additional articles. Afterwards, we performed backward snowballing looking at the references of all articles, obtaining four more studies. Two iterations of both forward and backward snowballing were carried out, as no new relevant articles were obtained after the second round.

The resulting 21 selected articles are classified into two categories depending on the scenario, where a database has been found. On the one hand, as a primary source [17]: the article is the main publication of the database or is directly related to it; and on the other hand, as a secondary source [4]: articles gathering existing databases from literature. From the primary source, 13 databases have been identified. There are more articles than databases since some articles provided newer or extended versions of the same databases. Articles from the secondary source provide 8 of the 13 databases, hence 5 databases are not considered in any of the gathering articles.

Answering RQ1, a total of 13 databases have been identified as a result of the systematic search: AGD [31, 32], ASD GD [33], AutDB [10, 32, 34–36], AutismKB [32, 36–38], BrainBase [39, 40], DBDB [41], EpideNovo [42], GeisingerDBD [14, 43], NPdenovo [44], PsyMuKB [45], SFARI Gene [9, 14, 28, 32], SysNDD [14, 46], and VariCarta [11, 32].

### Stage 2: Selection of databases

In this stage we filtered the 13 identified databases in order to select the pertinent ones to be used as ASD candidate gene sources. The results of the three dimensions (Accessibility, Currency, and Relevance) assessed for the databases are shown in Table 3.

**Table 3.** Dimensions and corresponding metrics assessed for the identified databases.

Database	Accessibility		Currency	Relevance
	Active link	Downloadable	Updated	Scoring method
AGD	No	No	No	No
ASD GD	No	Yes	No	No
**AutDB**	**Yes**	**Yes**	**Yes**	**Yes**
AutismKB	Yes	Yes	No	Yes
BrainBase	Yes	Yes	No	No
DBDB	No	No	No	Yes
EpideNovo	No	No	No	No
**GeisingerDBD**	**Yes**	**Yes**	**Yes**	**Yes**
NPdenovo	No	No	No	No
PsyMuKB	No	No	No	No
**SFARI Gene**	**Yes**	**Yes**	**Yes**	**Yes**
**SysNDD**	**Yes**	**Yes**	**Yes**	**Yes**
VariCarta	Yes	Yes	Yes	No

In bold, the four databases that are selected for further analysis: AutDB, GeisingerDBD, SFARI Gene, and SysNDD.

All databases with active link provided downloadable data. Two cases that required further analysis are NPdenovo and ASD GD. NPdenovo has an active web interface but the database cannot be navigated, nor can its information be downloaded, hence we consider the link to the database to be inactive. The second case is ASD GD, whose article states that the database was deposited in an open source repository. However, no such information could be obtained from this source at the date of writing. We also contacted for information but received no response. Since the article offers a publicly downloadable version as an excel file in the article, we consider the database to be inactive but the data downloadable.

For databases with inactive links, it was not possible to assess the downloadable and updated metrics; therefore, these metrics were evaluated as negative for those databases. All databases with active link provide the last update date, which was used for assessing the Currency dimension (updated metric). Databases passed this metric if the update date was greater than 2 years. Concrete last update dates within the 2-year window, and checked on 12th March 2025, are AutDB (March 2024), GeisingerDBD (November 2024), SFARI Gene (January 2025), SysNDD (February 2025), and VariCarta (December 2024).

We considered that a database provides a scoring method for ASD candidate genes if it provides a classification of the database genes with the purpose of prioritizing ASD candidate genes, as is the case for AutDB, AutismKB, DBDB, GeisingerDBD, SFARI Gene, and SysNDD. Note that GeisingerDBD has two modules of information but only one of them provides a scoring method, the loss-of-function (LoF) module, which is the one we consider for the analysis. VariCarta provides statistical information from the database content including rankings of genes, but these are not intended to be used for ASD candidate gene prioritization but as a statistical measure of the database content, as it is stated in the database’s documentation and in the associated publication. ASD GD provides additional information that could be used for prioritizing genes, but genes are not classified by the database. The remaining databases do not provide any scoring method.

In summary, 54% of the databases are accessible, 38% are updated and 54% are relevant for the task at hand. The four databases (31%) that verify all metrics are SFARI Gene, AutDB, GeisingerDBD, and SysNDD [Database website links are SFARI Gene (https://gene.sfari.org), AutDB (http://autism.mindspec.org/autdb/Welcome.do), GeisingerDBD (https://dbd.geisingeradmi.org), and SysNDD (https://sysndd.dbmr.unibe.ch).]. These are the databases potentially relevant to be used as ASD candidate gene sources, and provide the answer to RQ2. The deeper analysis of their data is conducted in the following section.

### Stage 3: Analysis of databases

In this stage we analyse the data of the four potentially relevant databases selected in the previous stage, namely AutDB, GeisingerDBD, SysNDD, and SFARI Gene. To do so, we assess the resulting dimensions of the DQ approach, Completeness and Consistency, to evaluate to which extent the information provided by the databases is complete and consistent. As described in section Stage 3: Analysis of databases, Completeness is assessed at schema and data level.

### Completeness at schema level

To evaluate the CSL, we examine whether the information provided by each database website covers the fields of interest specified in Table 2. Results of this examination are presented in Table 4.

**Table 4.** Fields of information covered by each database website.

	AutDB	GeisingerDBD	SFARI gene	SysNDD
**Variant information**				
Variant allele change	Yes	Yes	Yes	No
Variant type	Yes	Yes	Yes	No
CNV information	Yes	No	Yes	No
Family type	Yes	No	Yes	No
Inheritance information	Yes*	Yes	Yes	No
Parental transmission	Yes*	Yes	Yes	No
**Gene information**				
Inheritance pattern	No	No	No	Yes
Syndromic classification	Yes	No	Yes	No
Assoc. Disorders or Syndromes	Yes	Yes	Yes	Yes
**CSL (9 fields)**	**77.78%**	**55.56%**	**88.89%**	**22.22%**

* AutDB provides these fields of information only for rare variants and not for common ones, we assigned half of the total score.

SysNDD database is the only one not providing detailed information related to the reported variants from which the evidence is gathered, compromising traceability of the information. This database provides a summary with the evidence considered for the gene association, but no specific details or links are provided. The remaining three databases provide the reported variant details regarding allele change, variant type, and also inheritance information, as well as the parental transmission when the variant is inherited. Only two of them, AutDB and SFARI Gene specify the family type associated to the reported variant. In addition, these two databases provide a specific module on CNV evidence.

Regarding gene information, SysNDD is the only database providing information on the inheritance pattern of each gene. All four of them provide the associated disorders or syndromes for each gene and only AutDB and SFARI Gene provide the syndromic classification for their genes.

With this information we computed the CSL metric by applying equation (1). Percentages are contained in Table 4.

### Completeness at data level

The CDL metric is assessed considering the ASD candidate genes of each database. For databases specific of ASD, i.e. SFARI Gene and AutDB, all genes are considered. GeisingerDBD is filtered by the ‘Autism’ column, considering those genes marked as associated to ASD. As previously specified, we consider the genes contained in the LoF module, which is the one providing a scoring method. For SysNDD database, we filter the data by the ‘entities disease ontology name’ column, considering the ‘autism spectrum disorder’ phenotype but also all phenotypes containing the prefix autis-. With this filtering we cover phenotypes related to genes associated with syndromic autism [47], such as ‘Intellectual developmental disorder with autism and macrocephaly’.

**Figure 4.**
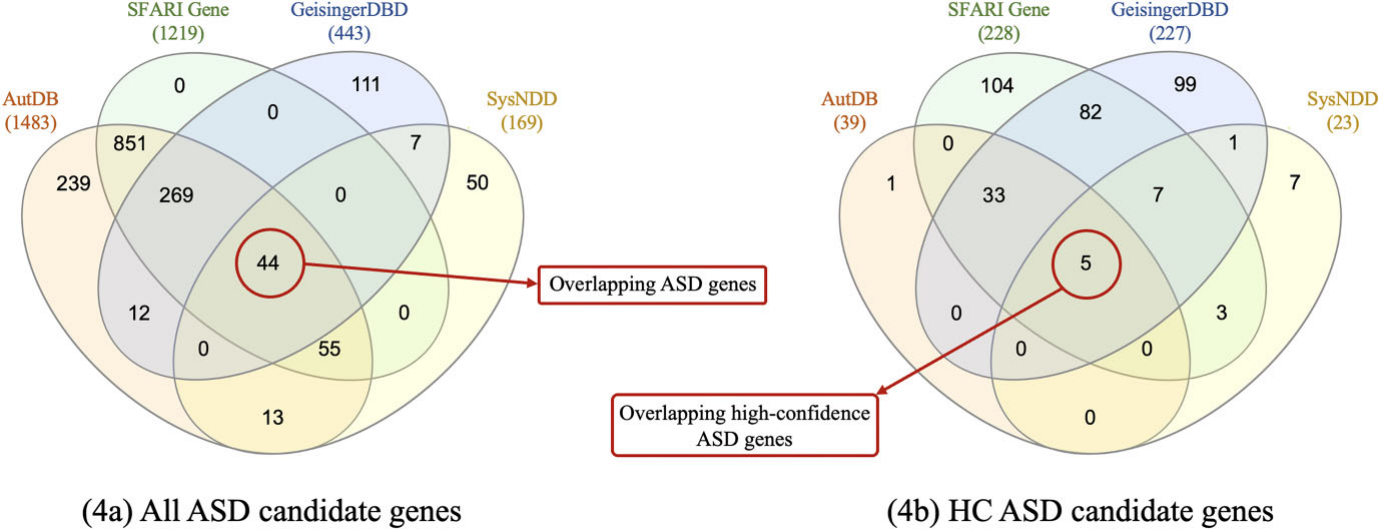
Distribution of genes among the four databases. These representations support the CDL—Fig. 4(a)—and consistency—Fig. 4(b)—metrics.

Figure 4(a) shows the distribution of ASD candidate genes found in all databases. SFARI Gene is the only database whose every gene is reported in at least one of the other databases. This is mainly because AutDB was licensed to SFARI Gene in 2012, hence all genes in SFARI Gene are also reported in AutDB. However, genes are classified differently in the two databases according to each scoring method. AutDB, GeisingerDBD, and SysNDD have a subset of ASD candidate genes reported exclusively in their data, representing the 16%, 25%, and 30% of all their ASD candidate genes, respectively. A total of 1650 ASD candidate genes are gathered from all four databases, of which only 44 (∼3%) are shared by all four of them. The list of 44 overlapping ASD candidate genes and their classification in each database can be seen in Supplementary file 1.

The results of applying equation (2) are shown in Table 5. The database containing the highest percentage of ASD candidate genes is AutDB, while the database with the lowest is SysNDD.

**Table 5.** Results for the CDL metric.

	AutDB	GeisingerDBD	SFARI gene	SysNDD
Num. ASD candidate genes	1483	443	1219	169
CDL (1650 unique genes)	89.88%	26.85%	73.88%	10.24%

Note that the total number of unique ASD candidate genes is not equal to the total number of genes provided by all databases given the data overlap.

### Consistency

The Consistency dimension is assessed considering the HC ASD candidate genes. The scoring method categories and where to find their definitions and criteria for each database is provided in Table 6.

**Table 6.** Scoring methods information.

Database	Scoring method categories	HC categories	Scoring method information
SFARI Gene	Category 1, Category 2, Category 3, Category S (syndromic)	Category 1	The scoring method criteria is explained in the database website [70]. SFARI Gene genes are categorized into syndromic (‘S’) or nonsyndromic, with ‘S’ genes linked to additional traits beyond ASD and ‘#S’ indicating independent evidence for idiopathic ASD. Nonsyndromic genes are classified into three categories: Category 1 (high confidence, ≥3 de novo likely gene-disrupting mutations or genome-wide significance), Category 2 (strong candidate, 2 de novo mutations or replicated GWAS evidence), and Category 3 (suggestive evidence, 1 de novo mutation or limited association data).
AutDB	Stars ranking: 5 to 1 stars, from highest to lowest confidence.	5 stars (*****)	The scoring method criteria is described in [29]. More details can be found in the database website [71]. AutDB genes are ranked according to the total strength of evidence gathered for the individual variants within each gene, which are manually annotated based on factors such as mode of inheritance, variant frequency and type, functional impact, genetic association significance, family structure, and zygosity.
GeisingerDBD	AR (Autosomal Recessive), Tier 1, Tier 2, Tier 3, Tier 4	AR and Tier 1	The scoring method criteria is explained in [43], and also specified in the database website [72]. GeisingerDBD genes are classified into four tiers based on the number of de novo pathogenic loss-of-function (pLOF) variants: Tier 1 (≥3 variants, HC), Tier 2 (2 variants), Tier 3 (1 variant), and Tier 4 (no variants), with autosomal recessive genes also included in Tier 1.
SysNDD	Definitive, Moderate, Limited, Refuted	Definitive	The scoring method criteria is explained in the database website [73]. SysNDD genes are classified into three categories based on the strength of genetic and clinical evidence linking them to intellectual disability and NDDs: Category 1 (‘Definitive’) includes genes with strong frequency, genetic, and/or clinical evidence (e.g. ≥10 de novo cases or equivalent criteria, such as ≥ 3 patients with de novo variant and variant recurrence); Category 2 (‘Moderate’) includes genes with moderate evidence (e.g. recurrent variants in smaller cohorts or functional support); and Category 3 (‘Limited’) includes genes with limited evidence from genetic or clinical findings, while genes not directly linked to NDDs are tagged as ‘n.a.’ (not applicable).

For each database: the scoring method categories, the categories providing the HC genes, and where to find the explanation of the scoring method criteria.

A ubiquitous criterion for a HC ASD candidate gene is the presence of at least three or more de novo LoF variants. However, scoring methods differ between databases when considering additional criteria, such as autosomal recessive inheritance, family structure, or syndromic classification.

To obtain the HC ASD candidate genes, we filtered by the corresponding level, specified in the third column of Table 6. In Fig. 4(b), we can see the distribution of HC ASD candidate genes. The total number of unique HC ASD candidate genes provided by all databases is 342, with SFARI Gene being the database with the highest number of HC ASD candidate genes, with 234 genes.

Only five ASD candidate genes are classified as HC by the four databases, representing a value of 1.5% for the CNS metric (equation 3). It is noteworthy that each database provides an exclusive set of HC genes, i.e. nonoverlapping with any of the other databases.

Percentages for Completeness gathered in Tables 4 and 5, and the percentage of Consistency provided in the preceding paragraph provide the results to answer RQ3.

## Discussion

### Summary of findings

In this study, we conducted a Systematic Mapping Study in order to identify databases containing information on ASD candidate genes. We further selected and analysed the potentially relevant databases to be used by clinicians as sources of information for genetic testing and counselling for ASD patients.

As a result of the Systematic Mapping Study, we obtained 21 articles from which 13 databases have been identified. Afterwards, we assessed the Accessibility, Currency, and Relevance dimensions, with 54% of the databases verifying accessibility, 38% currency and 54% relevance. The four databases (31%) verifying all metrics and thus selected as potentially relevant were SFARI Gene, AutDB, GeisingerDBD, and SysNDD. SFARI Gene and AutDB are ASD genetic resources while GeisingerDBD and SysNDD also encompass other NDDs. Data from these four databases was then assessed for the Completeness and Consistency dimensions. SFARI Gene has the highest CSL percentage (88.89%) while AutDB has the highest CDL percentage (89.88%), meaning SFARI Gene is the one covering more ASD information fields but AutDB being the database with the highest number of ASD candidate genes. SysNDD is the database with the lowest percentage values, being the least complete database at both schema and data levels.

Regarding Consistency, which is measured considering the HC genes, from a total of 342 HC genes gathered by all four databases there are 5 HC genes shared by all of them, resulting in a percentage value for consistency of 1.5%.

### Implications

Results of RQ1 include the 13 databases identified through our Systematic Mapping Study. While other studies report on ASD genetic databases [14, 32], they lack the comprehensive scope of our work, as they do not follow a systematic search methodology. Most tend to focus on widely known and recently updated databases [9, 11, 31, 35, 38, 39, 43, 46], whereas with a systematic approach a broader range is captured, including lesser-known resources [33, 41, 42, 44, 45]. Even though many of the identified databases are outdated or inactive, their documentation and methodologies remain valuable. Understanding the current landscape and prior efforts in building ASD genetic databases is crucial, since it ensures new developments are informed by existing work and avoid unnecessary duplication.

Results of RQ2 are obtained by filtering the 13 databases based on Accessibility, Currency, and Relevance to identify those most suitable for ASD candidate gene identification. While many ASD genetic databases have emerged over time, most are now inaccessible or outdated, limiting their practical use [31, 33, 41, 42, 44, 45]. As an example of the clinical implications of consulting outdated data given the time evolution of genomic research, consider the following two studies. Wright et al. [48] reanalysed genome information from 1133 families, discovering new genetic diagnoses through updated data and novel candidate genes. García et al. [49] highlighted the impact of evolving variant relevance in a case study on Early Onset Alzheimer’s Disease. These examples reinforce the need for current, reliable databases to ensure accurate ASD genetic testing and counselling.

Another key factor in assessing ASD gene databases is the presence of a gene scoring method, which we evaluated under the Relevance dimension. A tiered classification system is widely recommended [50], as genes vary in the strength of evidence linking them to ASD. Only one database, VariCarta, was excluded based on Relevance, despite meeting Accessibility and Currency criteria. Focused on variant-level evidence, VariCarta includes any gene with an ASD-associated variant, reporting over 200 000 genes without ranking or classification. This lack of stratification makes it unsuitable for directly identifying ASD candidate genes. However, its updated and accessible nature still makes it useful for specific inquiries such as variant effects.

Results of RQ3 show that the ASD-specialized databases, SFARI Gene, and AutDB, have the highest CSL. In contrast, SysNDD provides limited variant-related data, which interferes with traceability of information, but uniquely includes gene inheritance information. At the data level, AutDB reports the most ASD candidate genes (1483), yet GeisingerDBD and SysNDD, despite not being ASD-specific, contribute 168 unique genes absent in both SFARI Gene and AutDB (Fig. 4a). Notably, only 44 of the 1650 total unique ASD candidate genes (∼3%) are shared by the four databases. These results evidence the data dispersion problem previously mentioned, and also highlight the value of considering multiple databases, even those with fewer genes.

Finally, in terms of Consistency, we observed only a 1.5% overlap among HC ASD genes across all four databases, with 5 out of 342 genes being consistently classified. This result aligns with findings in [14], which reported 3.7% overlap for broader NDDs including ASD. The main reason for this low consistency is that scoring criteria differ between databases, which results in a different set of conditions for classifying a gene as HC. In addition, the body of evidence considered for evaluating a gene can also differ depending on the sources of information considered by each database.

Furthermore, many genes do not appear in all databases, as seen in the little overlap for the ASD candidate genes and discussed previously in the completeness metric, making consistent classification impossible. To isolate the effect of scoring criteria alone, we analysed the 44 ASD candidate genes that appear in all four databases (see Fig. 4a). Notably, 30 of these 44 genes are classified as HC by at least one of the databases, but only 5 are classified as HC by the four of them (see the 30 HC ASD genes in Supplementary file 1). This yields a higher but still low consistency rate of 17% (5/30), demonstrating the important effect of the differences in scoring method criteria and the strong need to work towards a standardized scoring method shared by all databases. Although there is already research being conducted in this direction, such as the EAGLE score [50], which aims to standardize gene classification (currently adopted by SFARI Gene), inconsistencies will persist without a unified scientific evidence base.

Completeness and consistency metrics results demonstrate that relying on a single database may lead to missed diagnoses or suboptimal counselling, as essential information such as HC ASD genes is spread and inconsistent across multiple sources. For the same reasoning, curation of gene lists from databases will result in a more comprehensive gene set if considering the union of HC genes, rather than the intersection, as databases may complement each other through additional evidence. Such integrated lists could also be used for developing a unified database of ASD genes, though translating scores to a standardized scoring criteria.

For a more comprehensive comparison, we cross-referenced the 30 HC ASD genes (identified across the four analysed databases) with two non ASD-specific but widely known resources: ClinGen [51], a curated resource defining the clinical relevance of genes and variants for precision medicine and diagnostics, and Gene2Phenotype [52], which presents comprehensive, scientifically supported gene–disease models that have been curated from the literature by experts. From the 30 HC ASD genes, 11 genes are classified as Definitive by ClinGen’s Intellectual Disability and Autism Gene Curation Expert Panel. Of these, four genes—ANK2, CHD8, FOXP1, and CNOT3—are recognized as HC by all four databases. Notably, NLGN4X and TRRAP are considered HC solely in SFARI Gene and SysNDD, respectively. The remaining five Definitive ClinGen genes—TBR1, PTCHD1, SHANK2, TANC2, and CUL3—are marked as HC by at least one database. Comparing with the Development Disorders Panel of Gene2Phenotype resource, 19 of the 30 genes are also reported in the panel. Considering only the genes related to autism, TBR1, FOXP1, and PTCHD1 are classified as Definitive, and CHD8, SHANK2, NLGN4X, and TRRAP as Strong.

Furthermore, a recent study [15] screened SFARI Gene, AutDB, and ClinVar to identify genes potentially specific to nonsyndromic ASD. Of the 20 genes proposed, only CHD8 and PTEN overlap with the 30 HC ASD genes, given that they follow a different procedure for selecting relevant genes based on the number of hits.

Given the high genetic heterogeneity of ASD, numerous genes and signalling pathways have been related to its pathogenesis. According to [53], dysregulation of both the mTOR and Wnt/β-catenin signalling pathways has been linked to ASD through their roles in neurodevelopment and synaptic function. The mTOR pathway, which is essential for synaptic protein synthesis, has been liked to variations in multiple genes, such as TSC1/TSC2, PTEN, NF1, EIF4E, and NLGN genes. Similarly, alterations in the Wnt/β-catenin signalling pathway, critical for central nervous system growth, have been associated with variants in genes such as CHD8, FZD9, WNT2, BCL9, and CTNNB1. Notably, PTEN, NF1, and CH8D genes are classified as HC by all four databases; TSC2 is HC according to AutDB, GeisingerDBD, and SFARI Gene; TSC1 and CTNNB1 are HC according to both GeisingerDBD and SFARI Gene; NLGN3 is HC according to both SFARI Gene and SysNDD, and NLGN2 and NLGN4X are HC according to SFARI Gene.

Finally, we emphasize the ongoing need of identifying ASD candidate genes. The value of the growing whole genome sequencing data depends largely on the insights derived from structured bioinformatic analyses, which require clear frameworks and guidance. Curated gene lists play a vital role in extracting meaningful genetic information, not only for enhancing diagnostic accuracy, but also for basic research, specifically for studying the association between genes and disease occurrence. To support this, we compiled an exhaustive list of ASD candidate gene databases from the literature and identified those most relevant for clinical and research use. Our comparative analysis revealed that while these databases vary in completeness, each offers unique clinical insights. The study also highlights the widespread dispersion of data and the absence of a standardized scoring system, which may result in overlooked or potentially misleading clinical interpretations. To further support clinicians and researchers, we propose the Checklist for ASD Candidate Gene Database Usage (Supplementary file 2), which translates our evaluation dimensions (Accessibility, Currency, Relevance, Completeness, and Consistency) into actionable steps for selecting and using these databases effectively.

### Limitations

Here, we explain the limitations of this study and how they were mitigated.

On the selection procedure of potentially relevant databases addressed by RQ2, the Relevance dimension imposes a rigorous metric, which is the presence of a scoring method. There is a threat of discarding relevant databases by imposing such a rigorous metric. However, this criterion cannot be abandoned, since it is aligned with the objective of this study and is chosen to ensure that high-trust information is considered, since it allows to prioritize the most relevant genes. The database affected by this metric is the case of VariCarta, which has already been discussed.

For the calculation of the CSL metric, the selection of fields to be considered is extracted from the literature (see Table 2), and then matched with the information provided by the databases. However, other fields also offered by the databases, such as information on protein interaction, could be potentially useful. This information would complement the richness of the study but does not affect the results presented here.

Additionally, the reliability of databases may be affected by biases. For instance, most genetic studies disproportionately focus on European ancestry populations, limiting the detection of population-specific risk genes and reducing the generalizability of the evidence. Databases also rely on published literature, which is subject to publication bias, and present differing curation practices, such as variable inclusion criteria and evidence weighting. These factors can lead to inconsistencies in gene scoring, favouring genes that are more extensively studied rather than those with stronger supporting evidence.

Finally, we highlight the following issues in the ASD genetics research domain. First, the winner’s curse [54, 55], a critical issue in genetic association studies, where initial studies overestimate effect sizes due to statistical fluctuations or selection bias, leading to replication problems such as finding a weaker effect or failing to replicate the result at all in later replication studies. The existence of studies with potential statistical biases due to the winner’s curse raises concerns about the rigour of data collected in the genetic databases analysed in this study. Second, ASD is characterized by being highly heterogeneous, with gene discovery patterns varying significantly depending on phenotypic severity, comorbidities, sex, and ancestry differences [5, 56, 57], which makes it more challenging to determine HC genes. It is noteworthy that SFARI Gene deals with these two issues. As previously mentioned, this database incorporates an additional score, the EAGLE score [50], which considers the replication of the evidence over time as a critical criterion for classifying a gene as HC. Moreover, SFARI Gene belongs to the The Simons Foundation, which leads the SPARK study. This study has conducted research on the variability of ASD gene discovery patterns [57–59], tackling the second issue mentioned before.

## Conclusions

This study offers a comparative view of multiple ASD-related gene databases for ASD candidate gene identification. We have compiled a comprehensive list of databases providing ASD candidate genes from the scientific literature by conducting a Systematic Mapping Study. We then applied a DQ approach to select those most relevant to be used as ASD candidate gene sources, namely SFARI Gene, AutDB, GeisingerDBD, and SysNDD, and conducted a comparative analysis of their data.

Our study shows that the four potentially relevant databases differ in terms of completeness, yet each contributes unique insights into ASD candidate genes. Notably, we found inconsistencies in how these databases classify HC ASD genes, stemming from variations in scoring methods and the scientific evidence each considers. This has important implications for genetic testing and counselling, as relying on a single database could lead to differing conclusions for the same clinical scenario. We highlight the value of each database in clinical practice and bioinformatic research, and advocate for their collective use to minimize the risk of missed diagnoses or misinterpretations caused by incomplete or overlooked information. Opportunities for improvement are identified in terms of scoring method standardization and the centralization of scientific evidence. This study contributes to ongoing ASD research by assessing the quality and reliability of ASD genetic databases to support more robust identification of ASD candidate genes, ensuring more accurate clinical interpretations and research advancements.

## Supplementary Material

baaf067_Supplemental_Files
