# Supplementary material for: Review of autism spectrum disorder databases for the identification of candidate genes
Source: Database (Oxford). 2025 Oct 15;2025:baaf067. doi: 10.1093/database/baaf067 (PMC12527254; doi:10.1093/database/baaf067)
Supplement: baaf067_Supplemental_Files [file baaf067_supplemental_files.zip › Supplementary_file_2.docx]

**Checklist for ASD Candidate Gene Database Usage**

☐ Confirm the database is **currently accessible** and not discontinued or inactive, and data is **downloadable**.

☐ Verify that the database has been **updated recently** (within the last 12–18 months) to avoid outdated evidence.

☐ Check whether the database provides a **clear and transparent gene scoring** **method** or classification system (e.g., high-confidence, emerging evidence).

☐ Assess whether the database includes **variant-level information** and **traceable references** to the supporting literature.

☐ **Cross-check candidate genes across multiple databases** (e.g., SFARI Gene, AutDB, GeisingerDBD, SysNDD) given the low overlap of high-confidence genes across all sources.

☐ **Document differences in gene classification**; inconsistencies often arise from variation in scoring criteria and underlying evidence.

☐ Verify that genes classified as high-confidence are **supported by ASD-specific evidence**, not only general neurodevelopmental findings.

☐ **Cross-reference with trusted external resources** (e.g., ClinGen or Gene2Phenotype) to see if the gene is considered Definitive or Strong for ASD.

☐ When compiling gene lists, use the **union of high-confidence genes** from multiple databases rather than the intersection to avoid missing evidence.

☐ For genes inconsistently classified, consider **manual review of the supporting literature** before clinical interpretation.
